# Commentary: The “efficacy window” phenomenon of daridorexant in insomnia treatment: a dose-dependent time-course meta-analysis

**DOI:** 10.3389/fphar.2026.1976948

**Published:** 2026-10-28

**Authors:** William V. McCall, Neil A. Burton, Björn Steiniger-Brach

**Affiliations:** 1 Department of Psychiatry and Health Behavior, Medical College of Georgia (MCG), Augusta University, Augusta, GA, United States; 2 Idorsia Pharmaceuticals Ltd., Allschwil, Switzerland

**Keywords:** approved label, daridorexant, DORAs, insomnia disorder, maintenance treatment, dose-response, long-term efficacy, benefit-risk

We read with great interest the recent article by Wang et al., “The “efficacy window” phenomenon of daridorexant in insomnia treatment: a dose-dependent time-course meta-analysis” published in *Frontiers in Pharmacology* (Wang et al., 2026). The authors provide an analysis of the efficacy and safety of daridorexant in insomnia disorder based on the published results of six randomized controlled trials (RCTs).

Although we recognize the merits of this meta-analysis which is the first to incorporate both the Japanese and Chinese Phase 3 studies alongside the global studies performed in the US and Europe, we believe that the conclusion of the manuscript is not fully supported by the totality of the evidence. The authors conclude that “In clinical practice, daridorexant 50 mg may be prioritized for its rapid onset of action; for long-term maintenance, 25 mg can be considered, although this dosage is associated with an elevated risk of adverse events of special interest, indicating that the benefits and risks must be carefully evaluated in clinical use.” This interpretation on long-term maintenance dosing may inadvertently overemphasize the pooled 3-month data available for 25 mg while underrepresenting the evidence supporting daridorexant 50 mg.

The available evidence does not support the conclusion that 25 mg should be the preferred dose for long-term maintenance. In the global Phase 3 studies (which were the only studies to contribute data beyond 1 month), the higher dose of 50 mg significantly improved both objective wake after sleep onset (WASO) and latency to persistent sleep (LPS), as well as subjective sleep parameters such as total sleep time (sTST) vs. placebo after 1 and 3 months of treatment. Importantly, 50 mg also significantly improved daytime functioning as assessed by the Insomnia Daytime Symptoms and Impacts Questionnaire (IDSIQ) at both month 1 and month 3 of treatment. With the lower dose of 25 mg, significant improvements versus placebo were observed on sleep parameters, while the effects on daytime functioning were less consistent than with 50 mg. Daridorexant was well tolerated at both doses, with rates of adverse events similar to placebo (Mignot et al., 2022).

In a subsequent dose-response analysis at month 1 and month 3 using data from the global Phase 2 and Phase 3 studies, the efficacy and safety of daridorexant were assessed vs. placebo at different doses. The data from this analysis support the use of the 50 mg dose as providing the greatest efficacy with no increased risk for adverse events as compared to the lower dose of 25 mg (Luyet et al., 2024).

The interpretation of the AESI findings also warrants caution. Although the Adverse Events of Special Interest (AESI) analysis reached statistical significance for the 25 mg subgroup and not for the 50 mg subgroup, the test for differences between dose subgroups was not statistically significant (P = 0.70) (Wang et al., 2026). Therefore, these findings do not establish a dose-specific increase in AESI risk with 25 mg compared with 50 mg. As Wang et al. acknowledge, the apparent signal may also reflect differences in sample size and duration of exposure across doses. Moreover, this signal rests on very small event counts (5 events among 531 patients at 50 mg vs. 14 events among 841 patients at 25 mg), with a wide confidence interval around the 25 mg estimate (RR 5.78 [1.52, 22.01]). The authors also note among their limitations that AESIs were not explored in detail and that further research is needed to clarify the risk profile associated with 25 mg (Wang et al., 2026), further underscoring the uncertainty of using this finding to inform a dose-specific recommendation for long-term maintenance.

In line with the evidence, the FDA and EMA have approved both the 25 and 50 mg doses. The US prescribing information recommends a dosage range of 25 mg–50 mg once nightly (Idorsia Pharmaceuticals Ltd. QUVIVIQ USPI, 2022). The European product information specifies daridorexant 50 mg once nightly as the recommended dose for adults with insomnia disorder, while 25 mg is only recommended based on clinical judgment in specific circumstances such as moderate hepatic impairment or co-administration with moderate CYP3A4 inhibitors (Idorsia Pharmaceuticals Ltd. QUVIVIQ SmPC, 2022).

As the authors of the current article note, only the 25 mg dose could be pooled from the two RCTs with data at 3 months, while the 50 mg dose could not because it was only included in one RCT at that timepoint. As a result, the 50 mg dose does not benefit from pooled estimates at month 3, despite the existence of positive 3-month efficacy data from the phase 3 RCT (Mignot et al., 2022) and the longer-term extension data which showed that daridorexant 50 mg achieved the most favorable improvements in patient-reported total sleep time and daytime functioning compared with placebo, that were sustained in the 9-month follow-up (Kunz et al., 2023). This limitation should not be interpreted as evidence favoring 25 mg for long-term maintenance. Importantly, demonstration of a statistically significant pooled effect for 25 mg in the absence of a pooled estimate for 50 mg does not constitute comparative evidence that 25 mg is preferable to 50 mg for maintenance treatment. The meta-analysis did not directly compare the relative efficacy of the two doses at 3 months. Taken together, the available evidence supports 50 mg as the dose associated with the greatest and most consistent clinical benefit. In fact, real-world studies provide further evidence supporting the effectiveness of daridorexant 50 mg during continued treatment in clinical practice (Palagini et al., 2024; Fernando et al., 2026; Hoxhaj et al., 2026; Palagini et al., 2026; Williams and Rodriguez-Cué, 2023). A recent consensus study among Italian sleep and insomnia experts also identified the statement that “daridorexant can be used at a dose of 50 mg/day, which has been shown to be the most effective in multiple clinical scenarios, unlike 25 mg/day, which is only useful in specific cases” as one of the most relevant recommendations for optimizing treatment (Ferini-Strambi et al., 2026).

Based on the above evidence, the recommendation of 25 mg for long-term maintenance in the meta-analysis by Wang et al. is not supported by comparative evidence and may lead to an incomplete interpretation of the evidence for daridorexant 50 mg, which is the EMA-recommended dose and the dose with the greatest clinical benefit on both sleep and daytime functioning with a favorable risk profile. It would be important to consider the sample sizes, follow-up duration, availability of pooled data, strength of evidence and the approved labels when making recommendations, to enable clinicians to make informed decisions in treating people with insomnia disorder.
